# Application of TOAST criteria, comorbidities and outcomes in patients with ischemic stroke: multicenter collaboration in the Dominican Republic

**DOI:** 10.3389/fstro.2026.1877826

**Published:** 2026-06-29

**Authors:** Ryna Then, Maria Muñoz, Cristina Ramos, Marcos Mota, Priscilla Sepulveda, Lisa José, Luis Suazo, Francisco Mendez, Yahaira Franco, Cosme González Villamán, Acela Gonell, Violiza Inoa, Gillian Gordon Perue, Manuel Colomé-Hidalgo, Nadja García

**Affiliations:** 1 Neurology Division, Jefferson Einstein Philadelphia Hospital, Philadelphia, PA, United States; 2 Thomas Jefferson University, Philadelphia, PA, United States; 3 Department of Medicine, Health Sciences Area, Instituto Tecnológico de Santo Domingo (INTEC), Santo Domingo, Dominican Republic; 4 Hospital Regional Universitario José María Cabral y Báez, Santiago, Dominican Republic; 5 Centro de Diagnóstico Medicina Avanzada y Telemedicina (CEDIMAT), Santo Domingo, Dominican Republic; 6 Department of Neuroendovascular Surgery, Clínica Corominas, Santiago de los Caballeros, Dominican Republic; 7 Unión Médica del Norte, Santiago, Dominican Republic; 8 Semmes-Murphey Clinic, Memphis, TN, United States; 9 University of Tennessee Health Science Center, Memphis, TN, United States; 10 Miller School of Medicine, University of Miami, Miami, FL, United States

**Keywords:** Dominican Republic, ischemic stroke, public health, stroke etiology, TOAST classification

## Abstract

**Introduction:**

Stroke is a leading cause of neurological-related death in the Caribbean. The Dominican Republic has among the highest stroke-related mortality rates in the Americas. We aimed to determine ischemic stroke etiologies, characterize comorbidities, and describe outcomes in the Dominican Republic using the TOAST (Trial of Org 10172 in Acute Stroke Treatment) classification.

**Methods:**

We analyzed a multicenter quality registry between January 2022 and October 2024. Statistical analyses included Mann–Whitney *U* and Kruskal–Wallis tests with Bonferroni correction, chi-square tests, and Spearman correlation.

**Results:**

The most common stroke subtype was undetermined etiology (54.8%), followed by large artery atherosclerosis (17.2%) and cardioembolism (16.5%). Hypertension (73.8%), diabetes mellitus (47.2%), and prior stroke (19.9%) were the most prevalent risk factors. Cardioembolic stroke had the highest median NIHSS score (6). Prior stroke was associated with increased odds of large artery atherosclerosis (OR 1.53, *p* < 0.05). Hyperlipidemia was associated with small vessel occlusion (OR 2.14), and atrial flutter/fibrillation with cardioembolic stroke (OR 19.6). Reperfusion therapy was administered to 162 patients (16.6%): intravenous thrombolysis (57%), thrombectomy (33%), and bridging therapy (10%). Inpatient rehabilitation access was limited. Physical therapy was the most common. Modified Rankin Scale scores at discharge increased significantly across all etiologies (*p* < 0.001), with the greatest worsening in cardioembolic stroke (+2).

**Conclusions:**

Most ischemic strokes were of undetermined etiology, with higher severity observed in cardioembolic stroke. Limited diagnostic workup, reperfusion therapies, and rehabilitation access highlight critical gaps in stroke care. These findings provide baseline data to inform targeted prevention and health system interventions in the Dominican Republic and similar settings.

## 1 Introduction

Stroke represents the second leading cause of death in the world (Naghavi et al., 2025) and the third cause of disability globally (Feigin et al., 2021). The burden of the stroke demonstrates significant epidemiological variation, particularly between high-income countries and low- to middle-income countries (LMICs). In all LMICs combined, stroke subtypes are distributed at approximately 63.4% for ischemic stroke and 31.1% for hemorrhagic stroke (Feigin et al., 2024). The global trajectory of the disease remains alarming; overall stroke mortality is projected to increase by nearly 50%, rising from 6.6 million deaths in 2020 to 9.7 million by 2050 (Feigin et al., 2023). Regionally, cardiovascular diseases—primarily ischemic heart disease and stroke—are the leading causes of death in the Americas, contributing substantially to premature mortality (Londoño et al., 2026). In Latin America and the Caribbean, stroke is the primary cause of death and disability of neurological etiology. The epidemiological burden in this region is worsening; according to the Global Burden of Disease (GBD) study, the Caribbean specifically demonstrated a concerning, increasing trend in the incidence of stroke between 2014 and 2021 (Hou et al., 2024).

Within this regional context, the Dominican Republic experiences a disproportionately severe stroke burden. According to 2021 statistics published by the Pan American Health Organization (PAHO), the Dominican Republic ranks among the countries with the highest stroke mortality rates in the Americas, preceded only by Haiti, Guyana, and Suriname. Furthermore, the nation registers some of the region's highest rates of years of life lost (YLL), total deaths, and years lived with disability (YLD) attributable to stroke (Pacheco-Barrios et al., 2022).

Addressing this high burden is complicated by local demographic and infrastructural challenges. The Dominican Republic is one of the most populated nations in the Caribbean region, with approximately 11,520,487 inhabitants (Worldometer. Dominican Republic Population, 2019), and hosts a massive annual influx of tourists that surpasses its resident population. The National Public Healthcare System consists of primary, secondary, and tertiary levels of care. While tertiary care includes highly specialized hospitals and subspecialty stroke care (Tolsa, 2017), public healthcare facilities generally lack access to first-line acute stroke treatments, such as intravenous thrombolysis and mechanical thrombectomy. The absence of these lifesaving and disability-sparing treatments in the public sector highlights a critical gap in the acute management of the disease.

Despite these alarming epidemiological trends and healthcare barriers, the Dominican Republic lacks comprehensive or representative data detailing the epidemiology and clinical characteristics of ischemic stroke. This study seeks to bridge this knowledge gap by assessing the national context and current standards of care during the acute phase of the disease. By providing essential epidemiologic data and characterizing etiology-specific insights, this research will establish a scientific foundation for effective stroke management. Ultimately, these findings aim to guide the development and implementation of targeted nationwide prevention and treatment strategies, equipping both public and private health centers with the evidence necessary to improve patient outcomes.

## 2 Materials and methods

### 2.1 Study design

This was a multicenter, retrospective, longitudinal observational study using prospectively collected data from the Stroke Care Quality Registry (RES-Q). Data were collected using the standardized RES-Q 3.0 form, a globally validated instrument designed to enhance stroke care quality. Participating hospitals submitted data for all stroke admissions; enrollment was consecutive for all patients meeting the inclusion criteria at participating centers. The registry captures data on both ischemic and hemorrhagic strokes; however, only ischemic strokes and TIAs were included in the present analysis. Data was abstracted by physicians who received standardized training in data collection.

### 2.2 Study setting and population

We retrospectively analyzed RES-Q data from January 2022 and October 2024. Only four hospitals from the Dominican Republic participated in the registry at the time of the study. The participating institutions included one public tertiary hospital currently undergoing certification as an Essential Stroke Center, and three private hospitals certified as Advanced Stroke Centers by the World Stroke Organization (WSO) (Lindsay et al., 2014). Stroke center certification requires ongoing participation in data collection and quality improvement initiatives, such as RES-Q. The certification of the first stroke center in the Dominican Republic in 2022 defined the start of our study period.

### 2.3 Participants

Eligible patients were adults (≥18 years of age) with a confirmed diagnosis of acute ischemic stroke (AIS) or transient ischemic attack (TIA). Exclusion criteria included incomplete documentation of predefined variables, hemorrhagic stroke, cerebral venous thrombosis, and stroke mimics.

Between January 2022 and October 2024, a total of 1,184 patients were screened. Following the exclusion of 208 cases (110 intracerebral hemorrhages, 53 subarachnoid hemorrhages, 30 stroke mimics, and 15 cerebral venous thrombosis), the final cohort comprised 976 patients. Within this cohort, 841 (86.2%) patients were diagnosed with AIS and 135 (13.8%) with TIAs (See Supplementary material S1).

### 2.4 Stroke etiology classification

Stroke etiology was classified according to the TOAST criteria by treating physicians at each participating center as part of routine clinical care. Because this study represents a retrospective analysis of prospectively collected registry data, etiology classification relied on diagnostic information available within the 2024 version of the RES-Q 3.0 form, without independent or centralized adjudication. Diagnostic workups were directed by local treating teams, leading to variations in the extent of investigation across sites. Notably, detailed diagnostic parameters—such as carotid Doppler ultrasound, Holter monitoring, and specific laboratory values—were not captured by the RES-Q registry at the time of data entry and were thus unavailable for this analysis. In cases with incomplete diagnostic data or inconclusive findings, treating clinicians systematically categorized the events as a stroke of undetermined etiology. This approach reflects the real-world nature of the registry, highlighting variations in access to advanced diagnostic modalities across hospitals, as well as the inherent constraints of the data collection tool.

### 2.5 Variables and data collection

A standardized data collection tool (See Supplementary material S2) was utilized to extract de-identified information from the RES-Q platform. While the RES-Q 3.0 platform captures a comprehensive array of stroke care metrics, our analysis was purposefully restricted to variables aligning with the study's predefined objectives. Extracted data encompassed sociodemographic characteristics (age and sex) and clinical comorbidities (hypertension, diabetes mellitus, hyperlipidemia, atrial fibrillation or flutter, smoking status, HIV, COVID-19, prior stroke or TIA, coronary artery disease, prior myocardial infarction, and congestive heart failure). Imaging parameters detailed the brain imaging modalities utilized, as well as the presence and location of arterial occlusions. Treatment data recorded the administration of intravenous thrombolysis, mechanical thrombectomy, or bridging therapy. Finally, neurological and functional outcomes were assessed using the National Institutes of Health Stroke Scale (NIHSS) and modified Rankin Scale (mRS) scores at both admission and discharge, alongside documentation of any specific rehabilitation therapies initiated during hospitalization.

### 2.6 Statistical analysis

Continuous data were assessed for normality using the Shapiro-Wilk test. Because all continuous variables exhibit a non-normal distribution, they are reported as medians with interquartile ranges (IQRs). Categorical variables are expressed as frequencies and percentages. Missing data were handled using a complete-case analysis approach.

Bivariate associations between risk factors and stroke subtypes were evaluated using crude (unadjusted) odds ratios (ORs) with 95% confidence intervals (CIs). Because multivariable adjustment was not performed, these crude ORs represent unadjusted associations and should not be interpreted as independent predictors. Due to the non-normal distribution of the continuous data, non-parametric tests were utilized for all inferential statistics. The Mann–Whitney *U* test and Kruskal–Wallis test were employed to assess differences in continuous and ordinal variables—specifically, National Institutes of Health Stroke Scale (NIHSS) and modified Rankin Scale (mRS) scores as well as mRS at 3 months —across two or more independent groups, respectively. Changes in NIHSS scores between admission and discharge were recorded as median differences. Categorical variables were compared using Pearson's chi-square tests of independence. Statistical significance was set at a two-sided *p*-value of < 0.05 (corresponding to a 95% confidence level), with analyses assuming an 80% statistical power. All statistical analyses were performed using IBM (IBM Corp., Armonk, NY, USA) SPSS Statistics software, and tables and figures were generated using Microsoft Excel (version 2501; Microsoft Corporation, Redmond, WA, USA).

### 2.7 Ethical considerations

This study was approved by the independent Ethics Committee of the Advanced Medicine Diagnosis and Telemedicine Center (CEDIMAT; Approval No. IRB00014368CEI-002). The requirement for informed patient consent was waived by the review board, as the research utilized data from an established quality improvement registry. The investigators report no conflicts of interest relevant to this article.

## 3 Results

### 3.1 Baseline characteristics and stroke etiology

Baseline characteristics of the study cohort are summarized in Table 1. The majority of patients were between 51 and 79 years of age (*n* = 626; 64.1%), and the cohort was predominantly male (57.2%). The most prevalent vascular risk factors were hypertension (73.8%), diabetes mellitus (47.2%), and a history of prior stroke (19.9%).

**Table 1:** Baseline patient characteristics, stroke severity (National Institutes of Health Stroke Scale) and Modified Rankin Scale (mRS) on Admission (*N* = 976).

Patient characteristics	*n* (%)	NIHSS on admission (median, IQR, CI 95%)^*^	mRS on admission (median, IQR, CI 95%)^*^
**Age**			
17–50	132 (13.5)	5 (7, 3.80–6.20)	0 (0)
51–79	626 (64.1)	5 (8, 4.37–5.63)	0 (0)
>80	218 (22.3)	6 (11, 4.54–7.46)	1 (2, 0.72–1.28)
**Sex**			
Male	559 (57.2)	5 (8, 4.33–5.67)	0 (0)
Female	417 (42.7%)	5 (8, 4.23–5.77)	1 (3, 0.682–1.318)
**Risk factors**			
Hypertension	720 (73.8)	5 (9, 4.33–5.66)	0 (1, 0–0.076)
Diabetes	461 (47.2)	5 (6, 4.455.55)	0 (1, 0–0.096)
Hyperlipidemia	70 (7.17)	4 (6, 2.57–5.43)	0 (1, 0–0.239)
Smoker	83 (8.50)	4 (7, 2.48–5.52)	0 (0)
Atrial fibrillation or flutter	73 (7.48)	5 (11, 2.46–7.54)	0 (2, 0–0.47)
HIV	2 (0.20)	15 (6, 6.68–23.32)	3 (5, 0–9.43)
COVID	7 (0.72)	3.5 (4, 0.30–6.70)	1 (1, 0.20–1.80)
Previous stroke	194 (19.9)	6 (8, 4.36–6.64)	0 (2, 0–0.29)
Coronary artery disease/previous myocardial infarction	68 (6.97)	5 (10, 2.59–7.41)	0 (1.5, 0–0.37)
Congestive heart failure	43 (4.41)	6 (9, 3.28–8.72)	0 (1, 0.31)
**Brain imaging types**			
Computed tomography	657 (67.32)	4 (7, 3.46–4.54)	0 (1, 0–0.081)
Computed tomography angiography	246 (25.20)	7 (10, 5.75–8.25)	0 (0)
DWI/FLAIR magnetic resonance imaging	48 (4.92)	4 (6.5, 2.16–5.84)	0 (0)
Magnetic resonance angiography DWI/FLAIR	4 (0.40)	4 (4.5, 0–8.41)	0 (1, 0–0.98)
Computed tomography perfusion	8 (0.81)	11 (13, 1.49–19.5)	0 (0)
No imaging documented	13 (1.33)	8 (14, 0.39–15.61)	0 (0)
**Occluded arteries**			
**Anterior circulation**			
Extracranial carotid artery	5 (0.51)	9 (6, 3.74–14.26)	2 (4, 0.0–4.94)
Intracranial carotid artery	29 (5.03)	11 (11, 7.0–15.0)	0 (1, 0.0–0.36)
M1	50 (5.12)	15 (7, 13.1–16.9)	0 (0)
M2	31 (3.18)	15 (8, 12.2–17.8)	0 (0)
M3	19 (1.95)	9 (7, 5.85–12.15)	0 (3, 0.0–1.39)
Anterior cerebral artery	6 (0.61)	13 (15, 1.0–25.0)	0 (0)
**Posterior circulation**			
Vertebral artery	10 (1.02)	1 (8, 0.0–6.23)	0 (0)
Basilar artery	2 (0.20)	20 (18, 0.0–44.95)	0 (0)
P1	5 (0.51)	6 (5, 1.62–10.38)	0 (0)
P2	4 (0.41)	9 (4.5, 4.6–13.4)	0 (3.5, 0.0–3.43)

NIHSS, National Institutes of Health Stroke Scale; mRS, Modified Rankin Scale.

^*^Values are presented as median (interquartile range) and 95% confidence interval.

Admission stroke severity, as measured by the NIHSS, was comparable between male and female patients. However, advanced age was associated with more severe strokes and higher degrees of disability upon admission. No significant associations were observed between individual vascular risk factors and stroke severity. In contrast, baseline disability varied by sex, with female patients presenting with greater disability at admission.

Regarding neuroimaging, non-contrast computed tomography (NCCT) was the most frequently utilized initial modality, followed by computed tomography angiography (CTA). Admission stroke severity varied by imaging modality, with patients undergoing CT perfusion (CTP) and CTA presenting with more severe strokes. Conversely, admission disability levels remained consistent across the different imaging groups.

Anterior circulation occlusions were the most frequently observed vascular deficits. The middle cerebral artery (MCA) was the most commonly affected vessel, primarily involving the M1 segment, followed by the M2 segment and the intracranial internal carotid artery (ICA). Within the posterior circulation, vertebral artery occlusions were the most common, followed by posterior cerebral artery (PCA) occlusions. Notably, occlusions involving the M1 and M2 segments were associated with the highest stroke severity at presentation.

Regarding stroke etiology, strokes of undetermined etiology comprised the largest proportion of the cohort (*n* = 535; 54.8%). Large vessel atherosclerosis was the second most common etiology (*n* = 168; 17.2%), followed by cardioembolism (*n* = 161; 16.5%) and small vessel disease (*n* = 85; 8.7%). Strokes of other determined etiologies accounted for the remaining 27 cases (2.8%; Table 2).

**Table 2:** TOAST classification in Dominican Republic (*N* = 976).

Stroke etiology	*n*	%
Large vessel atherosclerosis	168	17.2
Small vessel occlusion	85	8.7
Cardioembolism	161	16.5
Stroke of other determined etiology	27	2.8
Stroke of undetermined etiology	535	54.8

### 3.2 Associations between stroke subtypes and clinical factors

Table 3 shows the estimated odds associated with clinical risk factors across the different TOAST stroke etiologic subtypes. Patients with a history of prior stroke (OR: 1.53; 95% CI: 1.04–2.25, *p* = 0.03), hypertension (OR: 1.51; 95% CI: 1.01–2.29, *p* = 0.04), and diabetes mellitus (OR: 1.47; 95% CI: 1.056–2.05, *p* = 0.02) had a significantly increased likelihood of having large artery atherosclerosis.

**Table 3:** Relationship between TOAST stroke subtype and comorbidities.

Risk factors Odds ratio (95% Confidence Interval) *p*-Value					
Risk factors	Large vessel atherosclerosis	Small vessel occlusion	Cardioembolism	Stroke of other determined etiology	Stroke of undetermined etiology
Hypertension	1.519 (1.01–2.29) *p* = 0.047	0.92 (0.557–1.51) *p* = 0.737	3.21 (1.91–5.42) *p* < 0.0001	0.617 (0.433–0.880) *p* = 0.0076	0.71 (0.54–0.93) *p* = 0.012
Diabetes	1.47 (1.056–2.05) *p* = 0.023	1.62 (1.034–2.538) *p* = 0.035	0.947 (0.674–1.33) *p* = 0.754	1.028 (0.737–1.43) *p* = 0.869	0.731 (0.579–0.924) *p* = 0.009
Hyperlipidemia	1.047 (0.55–1.98) *p* = 0.887	2.14 (1.09–4.226) *p* = 0.028	1.63 (0.918–2.90) *p* = 0.096	0.644 (0.304–1.36) *p* = 0.251	0.684 (0.43–1.084) *p* = 0.106
Smoker	0.455 (0.23–0.92) *p* = 0.028	0.794 (0.356–1.769) *p* = 0.57	0.89 (0.5–1.57) *p* = 0.68	1.21 (0.72–2.04) *p* = 0.474	1.40 (0.94–2.06) *p* = 0.094
Atrial fibrillation or flutter	0.758 (0.371–1.550) *p* = 0.448	0.073 (0.004–1.193) *p* = 0.066	19.615 (11.69–32.90) *p* < 0.0001	0.0717 (0.0099–0519) *p* = 0.009	0.217 (0.122–0.387) *p* < 0.0001
HIV	0.6528 (0.04–12.18) *p* = 0.78	1.38 (0.07–25.78) *p* = 0.83	0.6919 (0.037–12.91) *p* = 0.81	2.006 (0.207–19.40) *p* = 0.55	3.01 (0.31–29.03) *p* = 0.34
COVID	3.292 (0.776–13.967) *p* = 0.106	0.7625 (0.043–13.391) *p* = 0.853	0.80 (0.098–6.567) *p* = 0.836	1.969 (0.392–9.884) *p* = 0.410	0.363 (0.073–1.81) *p* = 0.217
Previous stroke	1.53 (1.04–2.25) *p* = 0.03^*^	0.606 (0.32–1.16) *p* = 0.132	1.468 (0.987–2.182) *p* = 0.058	1.429 (0.966–2.114) *p* = 0.074	0.605 (0.449–0.817) *p* = 0.001
Coronary artery disease/previous myocardial infarction	0.795 (0.388–1.629) *p* = 0.531	0.503 (0.155–1.632) *p* = 0.253	2.865 (1.688–4.864) *p* = 0.0001	1.145 (0.60–2.17) *p* = 0.678	0.611 (0.378–0.987) *p* = 0.044
Congestive heart failure	1.04 (0.466–2.356) *p* = 0.932	0.263 (0.036–1.931) *p* = 0.189	3.491 (1.861–6.548) *p* = 0.0001	1.044 (0.459–2.373) *p* = 0.917	0.502 (0.272–0.929) *p* = 0.028

OR, odds ratio; CI, confidence interval 95%.

^*^Bolded values represent p < 0.05 for the Chi-Square of independence.

Hyperlipidemia (OR: 2.14; 95% CI: 1.09–4.23, *p* = 0.02) and diabetes mellitus (OR: 1.62; 95% CI: 1.03–2.54, *p* = 0.03) were significantly associated with higher odds of small vessel occlusion. Atrial flutter/fibrillation (OR: 19.61; 95% CI: 11.69–32.90, *p* < 0.0001), congestive heart failure (OR: 3.49; 95% CI: 1.86–6.54, *p* = 0.0001), hypertension (OR: 3.21; 95% CI: 1.91–5.42, *p* < 0.0001), and coronary artery disease (OR: 2.86; 95% CI: 1.69–4.86, *p* = 0.0001) were significantly associated with higher odds of cardioembolic stroke. HIV status (OR: 2.00; 95% CI: 0.21–19.40, *p* = 0.55), prior COVID-19 infection (OR: 1.96; 95% CI: 0.39–9.88, *p* = 0.41), prior stroke (OR: 1.42; 95% CI: 0.97–2.11, *p* = 0.07), and coronary artery disease (OR: 1.14; 95% CI: 0.60–2.17, *p* = 0.67) showed trends toward higher odds of stroke of other determined etiology, although these associations did not reach statistical significance. HIV infection (OR: 3.10; 95% CI: 0.31–29.03, *p* = 0.34) and smoking (OR: 1.40; 95% CI: 0.94–2.06, *p* = 0.09) were associated with increased odds of stroke of undetermined etiology; however, neither association reached statistical significance (Table 3).

### 3.3 Reperfusion therapies and clinical outcomes

Reperfusion therapies were administered to 162 patients, representing 16.6% of the total cohort (*N* = 976). Among these, most patients received intravenous thrombolysis (*n* = 92, 57%), followed by mechanical thrombectomy alone (*n* = 53, 33%) and combined thrombolysis and thrombectomy (*n* = 17, 10%; Table 4).

**Table 4:** Distribution of acute reperfusion therapies and changes in NIHSS and mRS from admission to discharge across ischemic stroke subtypes (*n* = 162).

Stroke etiology	Prevalence of treatment (%)	Only IVT	Only MT	IVT + MT	Pre-NIHSS^*^	Post-NIHSS^*^	Pre-mRS^*^	Post-mRS^*^
Large vessel atherosclerosis	14.9	17	6	2	5 (7, 3.94–6.06)	2 (5, 1.20–2.80)	0 (1, 0–0.154)	2 (2, 1.69–2.31)
Small vessel occlusion	12.9	11	0	0	5 (5, 3.94–6.06)	2 (3, 1.33–2.67)	0 (0)	1 (2, 0.55–1.45)
Cardioembolism	25.5	17	20	4	6 (11, 4.28–7.72)	3 (8, 1.64–4.36)	0 (1, 0–0.16)	2 (3, 1.51–2.49)
Stroke of other determined etiology	100	17	7	3	5 (8, 3.73–6.27)	2 (4, 0–4.14)	0 (0)	1 (2, 0–5.49)
Stroke of undetermined etiology	10.8	30	20	8	5 (8, 0–10.66)	2 (5, 0–9.45)	0 (0)	1 (3, 0–6)
Total	100	92	53	17				

IVT, intravenous thrombolysis; MT, mechanical thrombectomy.

^*^Values for mRS and NIHSS are expressed as median (IQR). Comparisons between pre- and post-values were conducted using the Mann–Whitney U test. All values were statistically significant.

Treatment distribution varied across stroke subtypes. The highest treatment prevalence was observed in strokes of other determined etiology, where all patients received reperfusion therapy (*n* = 27), most commonly thrombolysis (*n* = 17), followed by thrombectomy (*n* = 7) and combined therapy (*n* = 3). Large vessel atherosclerosis had a treatment prevalence of 14.9%, with thrombolysis being the most frequent modality (*n* = 17), followed by thrombectomy (*n* = 6) and combined therapy (*n* = 2). Small vessel occlusion (12.9%) was managed exclusively with thrombolysis (*n* = 11). Cardioembolic stroke demonstrated a higher treatment prevalence (25.5%) and accounted for the greatest number of thrombectomy cases (*n* = 20), followed by thrombolysis (*n* = 17). The lowest treatment prevalence was observed in strokes of undetermined etiology (10.8%), although this group contributed a substantial number of cases across all treatment modalities (thrombolysis *n* = 30, thrombectomy *n* = 20, combined therapy *n* = 8; Table 4).

Across all stroke subtypes, NIHSS scores significantly decreased from admission to discharge, indicating early neurological improvement (median reduction: −3; *p* < 0.001). In contrast, functional outcomes measured by mRS worsened at discharge compared to baseline across all groups, reflecting new disability following the acute event. The largest increase in mRS was observed in cardioembolic stroke and large vessel atherosclerosis (median change: +2; *p* < 0.001), while other subtypes showed a median increase of +1 (*p* < 0.001; Table 4).

Among the initial cohort, only 130 patients had available mRS data at 3-month follow-up. Patients treated with thrombectomy alone had the highest median mRS scores (5 at discharge and 4 at 3 months), whereas those treated with thrombolysis alone had the lowest median discharge mRS (median: 1). Combined therapy was associated with an intermediate median discharge mRS of 3. The effect size was greater in the thrombolysis-only group (0.09) compared to other treatment groups (0.023), likely reflecting differences in sample size. Three-month mRS data were not available for patients receiving combined therapy, precluding subgroup comparison at follow-up (Table 4).

Table 5 summarizes the distribution of ischemic stroke subtypes and their associated in-hospital mortality within the study population (*N* = 976). Large vessel atherosclerosis accounted for 168 cases and showed a mortality rate of 5.95% (10 deaths), corresponding to 1.02% of the total cohort. Small vessel occlusion represented 85 cases with the lowest mortality rate (2.35%, two deaths), corresponding to 0.20% of the total cohort. Cardioembolism comprised 161 cases and demonstrated a mortality rate of 9.32% (15 deaths), corresponding to 1.54% of the total cohort. Stroke of other determined etiology was less frequent (*n* = 27) but showed the highest subtype-specific mortality rate (25.93%, seven deaths), representing 0.72% of the cohort. Stroke of undetermined etiology was the most common subtype (*n* = 535), with a mortality rate of 2.99% (16 deaths) and accounting for 1.64% of the total cohort.

**Table 5:** Distribution of in-hospital mortality by stroke subtypes.

Stroke subtypes	*n*	Mortality rate *n* (%)	Proportion of the total cohort (*N* = 976) (%)
Large vessel atherosclerosis	168	10 (5.9)	1.02
Small vessel occlusion	85	2 (2.3)	0.20
Cardioembolism	161	15 (9.3)	1.54
Stroke of other determined etiology	27	7 (25.9)	0.72
Stroke of undetermined etiology	535	16 (2.9)	1.64

### 3.4 Access to rehabilitation therapies

**Figure 1:**
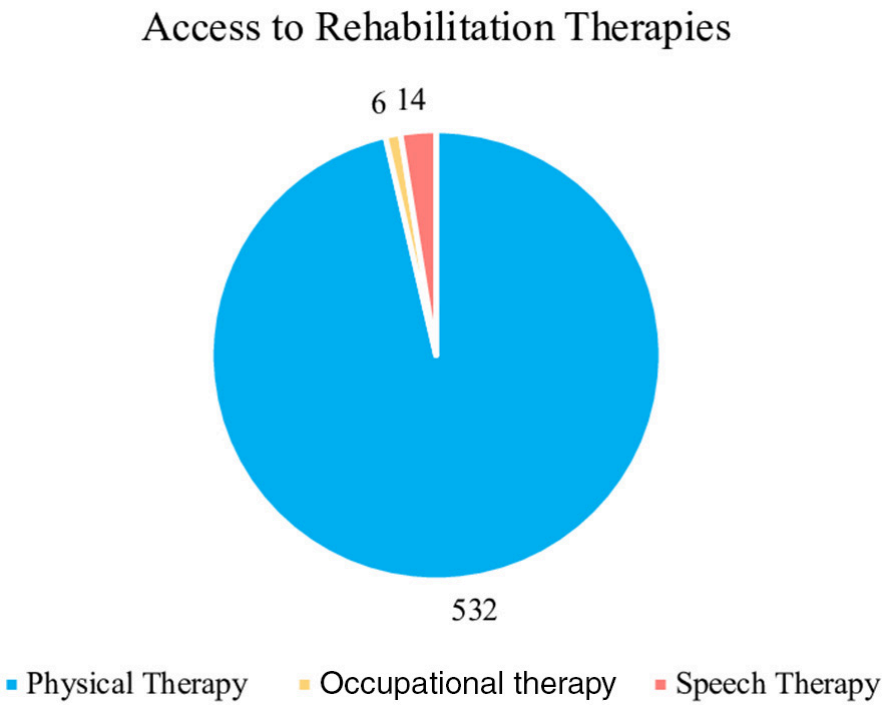
Access to rehabilitation therapies.

The distribution of rehabilitation services accessed by the study population is illustrated in Figure 1. Physical therapy (PT) was the most frequently provided service: 532 patients received PT, 214 were deemed not to require it, and 180 did not receive PT. Access to other rehabilitation modalities was infrequent, with six patients receiving occupational therapy (OT) and 14 receiving speech therapy (Figure 1).

## 4 Discussion

This multicenter study provides a comprehensive characterization of ischemic stroke etiology, patient characteristics, and risk factors across four stroke centers in the Dominican Republic. In our cohort, strokes of undetermined etiology and large-vessel atherosclerosis emerged as the most prevalent ischemic subtypes, followed by cardioembolism and small-vessel occlusion. These results offer a notable contrast to the Northern Manhattan Study (NOMAS) in the United States, where cardioembolic stroke was the most frequent subtype, followed by small-vessel disease and cryptogenic stroke (Gardener et al., 2020). In Martinique and Chile, small-vessel occlusion and cardioembolism represent the predominant etiologies (Lavados et al., 2007; Smadja et al., 2001). This discrepancy highlights the unique epidemiological landscape of the Caribbean and underscores the need for localized research to tailor stroke prevention and management strategies within the Dominican Republic and similar nations.

In our cohort, cardioembolic strokes and other determined etiologies were significantly associated with greater functional decline and higher in-hospital mortality. Atrial fibrillation (AF) remains a critical risk factor for cardioembolic events within this population, and in our analysis, AF was associated with increased morbidity and mortality rates. These findings highlight the potential prognostic relevance of etiological classification. However, given the observational design and the use of crude (unadjusted) odds ratios, these associations should be interpreted as descriptive patterns within our cohort rather than independent predictors of clinical outcome (Johansen et al., 2023). While strokes of other determined etiologies exhibited the highest case-fatality rates in our study, strokes of undetermined etiology contributed the largest absolute number of in-hospital deaths. This underscores the clinical challenge of managing cases where a definitive mechanism remains elusive, particularly in a resource-limited setting. Data regarding other determined etiologies remain relatively scarce in the existing literature. Given the diverse nature of these etiologies and their severe prognostic implications—such as the high mortality associated with cancer-related coagulopathy—a rigorous diagnostic workup is essential (Kim et al., 2022). However, our registry-based data did not capture specific cancer subtypes; consequently, we could not determine whether malignancy was the predominant mechanism in this group. This highlights the inherent constraints of a retrospective analysis of real-world registry data, where diagnostic intensity is dictated by local clinical practices and available resources.

Demographically, most stroke patients were between 51 and 79 years of age, reflecting the cumulative effects of aging on cerebrovascular health. Age remains an important non-modifiable risk factor, with stroke incidence doubling for each decade after age 55. Men were most frequently affected, which is consistent with global trends showing higher age-specific stroke incidence rates for men in this age bracket; however, women exhibited slightly higher incidences in the 35–44 and over-85 age groups (Goldstein et al., 2006). Notable sex differences also emerged regarding clinical severity upon presentation (Londoño et al., 2026). Women demonstrated a higher median mRS on admission compared to men, a finding that was statistically significant. These results contribute to supporting literature suggesting that older age at onset and potentially more severe events contribute to worse outcomes in women. Our data reinforces the need for sex-stratified analyses to better understand the biological and social determinants influencing stroke recovery in the Caribbean population (Renoux et al., 2017).

Hypertension, diabetes mellitus, and prior stroke emerged as the most prevalent risk factors in our population. Hypertension remains the leading population-attributable cause of cardiovascular disease worldwide, and its prominence in our study mirrors epidemiological patterns seen across the Caribbean (such as in Martinique) (Smadja et al., 2001) as well as broader Latin American trends where hypertension, high body mass index, smoking, and elevated fasting plasma glucose predominate (Pacheco-Barrios et al., 2022). Inadequate access to primary care, limited healthcare coverage, and systemic deficiencies in care quality continue to exacerbate the stroke burden in the Dominican Republic (Goldstein et al., 2006). Notably, the high prevalence of prior stroke in our study is of particular clinical concern, as recurrent events carry significantly higher mortality and treatment challenges (Alraddadi et al., 2025).

Analysis of stroke subtypes revealed strong etiologic links with traditional vascular risk factors. Hypertension, diabetes, and prior stroke were associated with large-vessel disease; diabetes and hyperlipidemia were linked to small vessel occlusion; and hypertension, atrial fibrillation/flutter, coronary artery disease, and congestive heart failure showed the strongest associations with cardioembolic stroke. In our cohort, smoking and HIV status were associated with higher odds for undetermined causes, though these did not reach statistical significance. Future longitudinal studies with larger sample sizes are necessary to clarify the impact of these emerging risk factors on the regional stroke burden, particularly in areas where HIV is prevalent (Figueroa, 2014). Specifically, the Dominican Republic is home to an estimated 46,000 (range: 33,000–59,000) HIV-1-infected individuals. This regional concentration is clinically significant, as HIV-positive individuals have been shown to have a substantially higher risk of any stroke subtype compared to HIV-negative controls (Gutierrez et al., 2017).

In terms of neuroimaging, non-contrast head CT (NCCT) was the most frequently performed modality, followed by CT angiography (CTA) of the head and neck. These findings align with current guidelines recommending immediate neuroimaging to facilitate rapid decision-making for thrombolytic therapy, with NCCT remaining essential for excluding intracranial hemorrhage (ICH) (Mendelson and Prabhakaran, 2021; Powers et al., 2019). Furthermore, our data reflect an increasing utilization of CTA to identify large vessel occlusions (LVOs). Current clinical standards increasingly champion the combination of NCCT and CTA as the minimum standard for the initial imaging of disabling stroke (Mayer et al., 2020). To ensure that the highest proportion of eligible patients has access to endovascular therapy (EVT), evaluation and treatment protocols must be streamlined. Despite these recommendations, our study revealed that only approximately 33% of patients underwent at least one screening imaging modality for LVO detection. This underutilization highlights significant systemic challenges within the Dominican Republic healthcare infrastructure. We hypothesize that knowledge gaps among providers, the absence of standardized stroke protocols, and the constraints of limited-resource settings contribute to this diagnostic deficit. Addressing these barriers is critical; without routine access to CTA, a substantial proportion of patients who could benefit from life-saving mechanical thrombectomy remain unidentified, further widening the gap between regional outcomes and international benchmarks (Mendelson and Prabhakaran, 2021).

Regarding anatomical distribution, the middle cerebral artery (MCA)—specifically the M1 segment—was the most frequently occluded vessel, followed by the intracranial internal carotid artery (ICA). Consistent with global clinical data, higher NIHSS scores were observed in patients with M1 and M2 occlusions, reflecting more severe clinical presentations and poorer prognosis. These findings reinforce the critical importance of early LVO recognition, as these patients represent a high-risk subgroup where targeted intervention is most impactful. Since the pivotal thrombectomy trials of 2015, EVT has become the standard of care alongside best medical therapy for LVO stroke. In our study, 16.6% of patients received acute reperfusion therapy, with intravenous thrombolysis (IVT) being the most common modality. This reperfusion rate aligns closely with recent multicenter data from Latin America, which reported an IVT rate of approximately 18%. While our 16.6% rate exceeds the averages of neighbors like Colombia (12%) and Mexico (9%), it remains below the 20%−25% thresholds consistently achieved in high-income networks like those in Austria and the USA (Kim et al., 2024). This parity with regional benchmarks suggests that while the Dominican Republic is progressing in its acute stroke response, significant systemic barriers—likely related to the aforementioned 33% CTA utilization rate—continue to limit access to mechanical thrombectomy for the majority of LVO patients (Dittrich et al., 2025).

Patients who received IVT alone exhibited more favorable functional outcomes and lower in-hospital mortality rates than those who underwent EVT. However, these outcomes must be interpreted with caution. Registry data consistently demonstrate that patients selected for EVT present with higher admission NIHSS scores, which are powerful predictors of poor prognosis (Salim et al., 2024). Thus, the observed differences in our cohort likely reflect the increased baseline severity and disability burden of the EVT subgroup rather than a lack of treatment efficacy. The therapeutic benefit of reperfusion in our cohort is best demonstrated by the Delta NIHSS (change from baseline). As previously validated by Agarwal et al. (2020) the percentage reduction in NIHSS serves as a more accurate marker of recanalization efficacy than raw final scores. Despite their more severe baseline presentations, all treated groups demonstrated significant neurological improvement following reperfusion. Our data confirm that even in high-severity LVO cases within the Dominican Republic, reperfusion therapy provides a measurable clinical benefit, further justifying the need to expand access to these interventions across both the public and private sectors.

A significant finding in our study was the notable underutilization of rehabilitation services, particularly occupational and speech therapy. This deficit likely reflects systemic structural and staffing limitations within the Dominican healthcare system rather than patient-level factors or a lack of clinical need. Such patterns are consistently reported across Latin America, where access to comprehensive stroke care remains fragmented. Data from Uruguay and Bolivia indicate that only 37%−65% of the population has access to acute stroke services and specialized post-stroke rehabilitation, even in nations with broader public health coverage. Data from Uruguay and Bolivia indicate that only 37%−65% of the population has access to acute stroke services and specialized post-stroke rehabilitation, even in nations with broader public health coverage. These gaps in the continuum of stroke care are particularly concerning given that early and multidisciplinary rehabilitation is essential for maximizing functional recovery and reducing long-term disability (Martins et al., 2019). The limited availability of these services in our cohort suggests that many Dominican stroke survivors may not be reaching their full recovery potential, further increasing the socioeconomic burden of the disease. Addressing this requires not only the implementation of acute protocols but also the expansion of specialized rehabilitation infrastructure and the training of dedicated allied health professionals (Pandian et al., 2020). At a regional level, the Latin America and Caribbean (LAC) region achieves only approximately 27.5% availability of rehabilitation services, a figure substantially lower than that observed in high-income regions (38.7%) (Owolabi et al., 2021).

Regional surveys further indicate that many patients experience delayed or absent access to rehabilitation following hospital discharge, while community-based remain insufficiently developed (Martins et al., 2021).

This study highlights the complex interaction between stroke etiology, severity, treatment access, and outcomes in the Dominican Republic. While acute therapies significantly improve neurological status, disparities in treatment utilization and rehabilitation access remain critical targets for improving stroke care and reducing long-term disability and mortality. Further research is needed to evaluate the prevalence of stroke risk factors across different regions of the country, where traditional risk factors may vary in distribution. It is imperative to develop stroke-specific prevention strategies aimed at improving education and awareness of cerebrovascular risk factors, enhancing recognition of stroke signs and symptoms, expanding access to care, and establishing infrastructure to support the needs of stroke patients. In addition, workforce training, resource recruitment, and etiology-specific treatment planning are essential to enable the development of targeted, life-saving interventions.

### 4.1 Limitations

This study has several inherent limitations that should be considered when interpreting the results. First, as a registry-based analysis, findings are geographically restricted to the Dominican Republic. Furthermore, the data are derived exclusively from the four hospitals (one public tertiary hospital and three private WSO-certified Advanced Stroke Centers) that participated in the RES-Q registry during the study period. Consequently, this cohort represents a multicenter, hospital-based sample rather than a nationally representative population. Because a standardized national denominator for stroke-capable hospitals and hospitalized ischemic stroke cases is unavailable, the exact proportion of national stroke patients captured by this cohort cannot be determined. Importantly, the overrepresentation of private centers in our sample likely introduces selection bias, limiting the generalizability of our findings to uninsured or socioeconomically vulnerable populations who predominantly rely on the public healthcare system.

Second, due to the retrospective nature of the data extraction and reliance on the RES-Q form, certain clinical details—such as confirming whether every patient received initial neuroimaging—could not be universally verified. Although rigorous standardization measures were implemented, including de-identified data collection, structured abstractor training, expert oversight, and periodic data quality reviews, residual information bias and variability in documentation practices across sites cannot be entirely excluded. Additionally, while mechanisms were in place to verify case capture and centers were encouraged to prospectively log all eligible cerebrovascular presentations, some degree of under-capture likely occurred. Thus, not all suspected stroke cases, including stroke mimics, presenting to participating hospitals may be fully represented.

Third, temporal changes in stroke systems of care—including evolving hospital resources, fluctuating staffing, and staggered stroke center certifications during the study period—may have influenced patient outcomes independently of the variables analyzed. Because our study did not account for these time-dependent factors, residual confounding related to ongoing improvements in stroke care delivery cannot be excluded. There is also a notable scarcity of long-term follow-up data, specifically regarding 3-month functional outcomes (mRS). This introduces a risk of attrition bias, as patients who returned for follow-up may systematically differ from those who did not. Consequently, findings regarding long-term functional recovery must be interpreted with caution.

Finally, several methodological constraints must be noted. Our statistical approach was limited to bivariate comparisons; therefore, the reported odds ratios (ORs) are crude (unadjusted) and do not account for potential confounding variables. As such, these associations should not be interpreted as independent predictors of clinical outcome. Additionally, our cohort included both acute ischemic stroke (AIS) and transient ischemic attack (TIA) cases. While this inclusion provides a comprehensive overview of acute cerebrovascular events, TIA cases differ significantly from AIS in clinical severity and prognosis. Because we did not perform a sensitivity analysis excluding TIA cases, this clinical heterogeneity should be considered when interpreting our findings.

## Data Availability

The raw data supporting the conclusions of this article will be made available by the authors, without undue reservation.
