# Supplementary material for: Application of TOAST criteria, comorbidities and outcomes in patients with ischemic stroke: multicenter collaboration in the Dominican Republic
Source: Front Stroke. 2026 Jun 29;5:1877826. doi: 10.3389/fstro.2026.1877826 (PMC13356938; doi:10.3389/fstro.2026.1877826)
Supplement: Supplementary file 1 [file Data_Sheet_1.PDF]

Supplementary Material

1 Supplementary Data

Supplement #1. Patient selection and screening.

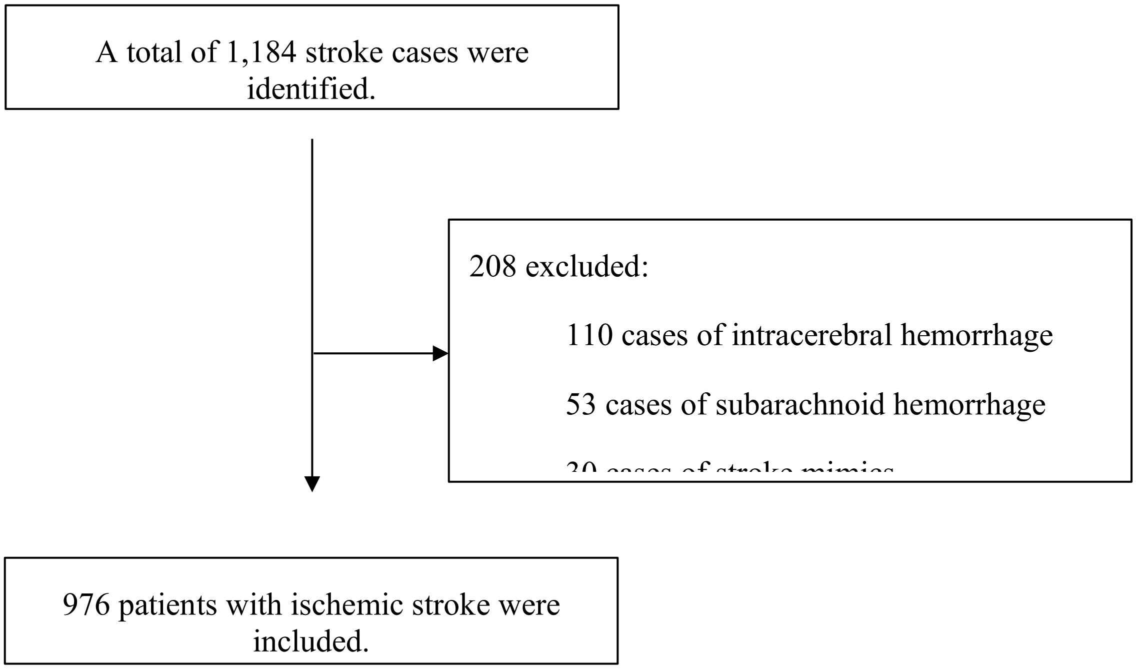

Supplement #2. Data collection tool.

Does the record have the TOAST criteria completed?

- Yes_______ No_______

Does the record include CT and MRI neuroimaging?

- Yes_______ No_______

Type of stroke

- Ischemic stroke ______
- Transient ischemic attack ______
- Intracerebral hemorrhage________
- Subarachnoid hemorrhage ______
- Cerebral venous thrombosis _______
- Stroke Mimicker-Other _______

Sociodemographic and clinical data

- Age _______
- Gender _______

Risk factors

- Hypertension ______
- Diabetes ______
- Hyperlipidemia ______
- Active smoker in the last 10 years ______
- Coronary artery disease ______
- Previous myocardial infarction ______
- Previous hemorrhagic stroke leading to hospitalization ______
- Previous ischemic stroke leading to hospitalization _____
- Atrial fibrillation or flutter ______
- HIV ______
- Hormonal contraceptives ______
- COVID-19 _____
- Congestive heart disease _____
- None ______
- Unknown ______
- Other: ______

Etiology

- Is the etiology known? Yes____ No_____
- Large artery atherosclerosis _______
- Small vessel occlusion or lacunar _______
- Cardioembolism ______
- Other determine etiology (hematological disorder, vascular dissection, etc.) _______
- Undetermine etiology ______

Neuroimaging used

- Head CT scan _____
- Head and neck CT angiogram____
- CT Perfusion _________
- Brain magnetic resonance (MRI) ______
- Brain magnetic angiogram (MRA)_____
- Cerebral Angiogram_____

Left	Right
MCA M1 Middle cerebral artery M1	MCA M1 Middle cerebral artery M1
MCA M2 Middle cerebral artery M2	MCA M2 Middle cerebral artery M2
MCA M3 Middle cerebral artery M3	MCA M3 Middle cerebral artery M3
Anterior cerebral artery	Anterior cerebral artery
PCA P1 Posterior cerebral artery P1	PCA Posterior cerebral artery P1
PCA P2 Posterior cerebral artery P2	PCA P2 Posterior cerebral artery P2
External carotid artery	External carotid artery

Left	Right
MCA M1 Middle cerebral artery M1	MCA M1 Middle cerebral artery M1
MCA M2 Middle cerebral artery M2	MCA M2 Middle cerebral artery M2
MCA M3 Middle cerebral artery M3	MCA M3 Middle cerebral artery M3
Anterior cerebral artery	Anterior cerebral artery
Internal carotid artery	Internal carotid artery
Basilar artery	Basilar artery
Vertebral artery	Vertebral artery

Scale scores

- NIHSS on admission __________
- NIHSS on discharge __________
- mRS on admission __________
- mRS on discharge __________

Did the patient receive physical therapy?

- Yes______
- No______
- Not required ______

If yes, did they receive

- Occupational therapy ______
- Speech therapy ______
- Physical therapy______
