# Supplementary material for: Application of TOAST criteria, comorbidities and outcomes in patients with ischemic stroke: multicenter collaboration in the Dominican Republic
Source: Front Stroke. 2026 Jun 29;5:1877826. doi: 10.3389/fstro.2026.1877826 (PMC13356938; doi:10.3389/fstro.2026.1877826)
Supplement: Supplementary file 2 [file Data_Sheet_2.PDF]

RES-Q Data for Life

3.0 Standard Data Collection Form

ALL STROKES: HOSPITALIZATION DATA

Denotes mandatory fields

Patient ID	Auto Generated (in the online form)	Date of admission	DD-MM-YY

ALL STROKES: ADMISSION DATA

Age	Years	Gender	☐ Male	☐ Female	☐ Other

Stroke while already hospitalized (select one)
- ☐ Yes
- ☐ No
- ☐ Unknown

Wake up stroke
- ☐ Yes
- ☐ No

If yes, time when patient went to bed: HH:MM

Date of admission	DD-MM-YY	Time of admission (hospital door)	HH:MM

Date of onset of stroke symptoms: DD-MM-YY
- ☐ Unknown

Time of onset of stroke symptoms: HH:MM
- ☐ No

Where was the patient first attended to at your hospital?
- ☐ Direct to CT/MR imaging suite
- ☐ Emergency department/casualty
- ☐ Outpatient clinic/facility
- ☐ Other department

Patient arrived to your hospital from
- ☐ From home/scene by EMS/ambulance → **Was the hospital pre-notified by EMS /ambulance** ☐ Yes ☐ No
- ☐ Home/scene by private/ own transportation
- ☐ Another hospital → **Name of the first hospital of admission**

Patient hospitalized in (day 1)
- ☐ ICU/Stroke unit
- ☐ Other monitored bed with telemetry
- ☐ Standard bed

Patient admitted under which department?
- ☐ Neurology
- ☐ Neurosurgery
- ☐ Critical care
- ☐ Internal medicine
- ☐ Other

ALL STROKES: HOSPITALIZATION DATA

Previous known history (select all that apply)

- [ ] Hypertension
- [ ] Diabetes
- [ ] Hyperlipidemia
- [ ] Active smoker in the last 10 years
- [ ] Coronary artery disease/ previous myocardial infarction
- [ ] Previous haemorrhagic stroke leading to hospitalization
- [ ] Atrial fibrillation or flutter (paroxysmal/persistent/permanent)
- [ ] Coronary artery disease/ previous myocardial infarction
- [ ] Congestive heart failure
- [ ] Hormonal contraception
- [ ] HIV
- [ ] Others
- [ ] None
- [ ] Unknown

Treatment before admission/event (select all that apply)

- [ ] Anti-diabetics
- [ ] Anti-hypertensives
- [ ] Aspirin (ASA)
- [ ] Cilostazol
- [ ] Clopidogrel
- [ ] Ticagrelol
- [ ] Ticlopidine
- [ ] Prasugrel
- [ ] Dipyridamol, slow release
- [ ] Warfarin
- [ ] Low molecular weight Heparin/Heparin
- [ ] Dabigatran
- [ ] Rivoroxaban
- [ ] Apixaban
- [ ] Edoxaban
- [ ] Statin
- [ ] None
- [ ] Other
- [ ] Unknown

'Glucose (first measurement in your hosp)* {Y(mmol/l)=X mg/dl * 0.18}

mmol/l

- [ ] Not done

LDL cholesterol (first measurement in your hosp) {Y (mmol/l) = X (mg/dl) / 38.7}

mmol/l

- [ ] Not done

Systolic Blood Pressure (first measurement in your hosp)

mmHg

Diastolic Blood Pressure (first measurement in your hosp)

mmHg

NIHSS on admission

Score

- [ ] Not done

Modified Ranking Scale (mRS) score

- [ ] 1
- [ ] 2
- [ ] 3
- [ ] 4
- [ ] 5

Unknown

First INR testing done?

- [ ] Yes, with point of care device
- [ ] Yes, sample sent to lab
- [ ] Not done
- [ ] Unknown

Glasgow coma scale

Value 3-15

Was the patient Covid positive?

- [ ] Yes
- [ ] No
- [ ] Not tested
- [ ] Recovered from COVID in last 6 months

IMAGING, DIAGNOSIS AND TREATMENT

Brain imaging type		
	☐	Non-Contrast CT
	☐	Non-Contrast CT + CT Angiography
	☐	Non-Contrast CT + CT Angiography + CT Perfusion → CT/ MR Perfusion deficit
	☐	MR DWI / Flair
	☐	MR DWI / Flair + MR Angiography
	☐	MR DWI / Flair + MR Angiography + MT Perfusion → CT/ MR Perfusion deficit
	☐	Imaging done at another hospital → Date of imaging: DD-MM-YYYY; Time of imaging: HH:MM
	☐	Not done

CT/ MR Perfusion deficit	
☐	Medial
☐	Anterior
☐	Posterior
☐	Carotid
☐	No, analysis not possible due to bilateral stenosis
☐	No deficit
☐	Not done

Date of imaging	DD-MM-YYYY	Time of imaging	HH:MM

Time of imaging	HH:MM

Old infarcts seen on the imaging (select all that apply)		
	☐	Cortical
	☐	Subcortical (basal ganglia, internal capsule)
	☐	Brainstream
	☐	None

Stroke type			
	☐	Ischemic Stroke	Complete section on page 4
	☐	Intracerebral Hemorrhage	Complete section on page 5
	☐	Transient Ischemic Attack (TIA)	Go to Post Acute section on Page 7
	☐	Subarachnoid Hemorrhage	Complete section on page 6
	☐	Cerebral Venous Thrombosis	Complete section on page 6
	☐	Stroke Mimics	Complete section on page 6
	☐	Undetermined (unknown stroke type)	Go to Post Acute section on Page 7

DIAGNOSIS AND TREATMENT

ASPECT score	

CTA/MRA occlusion	
	Yes
	No
	Not done

If arterial occlusion is present then select location (select all that apply)

Occlusion Location (Left)	Occlusion Location (Right)
MCA M1 - Middle cerebral artery M1	MCA M1 - Middle cerebral artery M1
MCA M2 - Middle cerebral artery M2	MCA M2 - Middle cerebral artery M2
MCA M3 - Middle cerebral artery M3	MCA M3 - Middle cerebral artery M3
Anterior cerebral artery	Anterior cerebral artery
PCA P1 - Arteria cerebri posterior P1	PCA P1 - Arteria cerebri posterior P1
PCA P2 - Arteria cerebri posterior P2	PCA P2 - Arteria cerebri posterior P2
Carotid artery extracranial	Carotid artery extracranial
Carotid artery intracranial	Carotid artery intracranial
Basilar artery	Basilar artery
Vertebral artery	Vertebral artery

Was the patient treated with IV Thrombolysis in your hospital — Yes / No

Treatment (select one)	
	Alteplase
	Tenecteplase
	Streptokinase
	Staphylokinase

Treatment dose (in mg)	

Bolus time	HH:MM

IV thrombolysis given in	
	CT/MRI room
	Stroke/ICU
	Emergency room
	Other

Antidote anticoagulant given	
	Yes
	No

Please select main reason for not doing thrombolysis	
	Already received IV Thrombolysis in other hospital
	Out of time window
	Mild deficit
	Consent not given
	Cost of treatment
	Transferred to other hospital for IV Thrombolysis
	Only Mechanical thrombectomy required
	TPA not available
	Others (please specify)

Transfer time (door out)	HH:MM

DIAGNOSIS AND TREATMENT CONTINUED

ISCHEMIC STROKE CONTINUED

Was the patient treated with Mechanical Thrombectomy in your hospital	☐ Yes	☐ No

If Yes:

Groin puncture time	HH:MM
Reperfusion time	HH:MM

TICI score
☐ 0
☐ 1
☐ 2A
☐ 2B
☐ 2C
☐ 3
☐ Occlusion was not confirmed

Procedure complications in thrombectomy
☐ None
☐ Vessel perforation
☐ Dissection
☐ Embolization to different vascular territory
☐ Haematoma at arterial access requiring transfusion
☐ Other

If No:

Please select main reason for not doing thrombectomy	
	☐ Already received IV Thrombolysis in other hospital
	☐ Out of time window
	☐ Mild deficit
	☐ Large vessel occlusion
	☐ Premorbid disability
	☐ Consent not given
	☐ Cost of treatment
	☐ Transferred to another hosp for Mechanical thrombectomy
	☐ MT facility not available in the hosp
	☐ Other

Transfer time (door out)	HH:MM

NOW COMPLETE POST ACUTE SECTION ON PAGE 7

INTRACEREBRAL HAEMORRHAGE

Bleeding volume (ml)	
Infratentorial source of bleeding	☐ Yes ☐ No
Source of bleeding found	☐ Yes ☐ No
ICH score (value 0-6)	
Presence of blood in the chambers	☐ Yes ☐ No

Reason for bleeding (select all that apply)	
	☐ Arterial hypertension
	☐ Aneurysm
	☐ Arteriovenous malformation
	☐ Anticoagulation therapy
	☐ Amyloid angiopathy
	☐ Other/not known

If neurosurgery was performed, select the type (select all that apply)	
	☐ Intracranial hematoma evacuation
	☐ External ventricular drainage
	☐ Decompressive craniectomy
	☐ Not required

NOW COMPLETE POST ACUTE SECTION ON PAGE 7

DIAGNOSIS AND TREATMENT CONTINUED

SUBARACHNOID HAEMORRHAGE

Hunt Hess score		
	☐	1 (lucid, mild headache, slight neck stiffness
	☐	2 (moderate to severe headache; neck stiffness; no neurologic deficit except cranial nerve palsy)
	☐	3 (drowsy; minimal neurologic deficit)
	☐	4 (stuporous; moderate to severe hemiparesis; possibly early decerebrate rigidity and vegetative disturbances)
	☐	5 (deep coma, severe neurological deficit)

Intervention (select all that apply)		
	☐	Endovascular (coiling)
	☐	Neurosurgical (clipping)
	☐	Ventricular drainage
	☐	Decompression craniectomy
	☐	Other procedure

NOW COMPLETE POST ACUTE SECTION ON PAGE 7

CEREBRAL VENOUS THROMBOSIS

Treatment		
	☐	Anticoagulation
	☐	Endovascular intervention (thrombectomy)
	☐	Endovascular intervention (local thrombolysis)
	☐	Neurosurgical treatment (decompressive craniectomy)
	☐	None

TRANSIENT ISCHEMIC ATTACK (TIA)

COMPLETE POST ACUTE SECTION ON PAGE 7

STROKE MIMIC

Final diagnosis (select one)		
	☐	Migraine
	☐	Seizure
	☐	Delirium
	☐	Electrolyte or metabolic imbalance
	☐	Functional disorder
	☐	Other

Was patient treated with IV thrombolysis		
	☐	Yes
	☐	No

Treatment		
	☐	Alteplase
	☐	Tenecteplase
	☐	Streptokinase
	☐	Staphylokinase

Bolus time	HH:MM
Dose (in mg)	

Antidote anticoagulant given?		
	☐	Yes
	☐	No

NOW COMPLETE POST ACUTE SECTION ON PAGE 7

POST ACUTE CARE

Patient hospitalized for more than 48 hours

☐ **Yes**	☐ **No, Patient transferred**	☐ **No, Patient died**
Complete the form on this page	Complete the form on page 9	Enter discharge date on page 9

Patient ventilated	
☐	Yes
☐	No
☐	Unknown

Venous Thromboembolism (VTE) Interventions

☐	Low Dose Unfractionated Heparin (LDUH)
☐	Low Molecular Weight Heparin (LMWH)
☐	Intermittent Pneumatic Compression Devices (IPC)
☐	Graduated Compression Stockings (GCS)
☐	Warfarin
☐	Venous Foot Pumps (VFP)
☐	Oral Factor Xa Inhibitor
☐	Asprin
☐	Others
☐	None

Carotid arteries imaging within 7 days after admission	
☐	Yes
☐	No

Symptomatic carotid stenosis	
☐	>70%
☐	50-70%
☐	No
☐	Unknown

Carotid endarterectomy performed or stenting within 2 weeks after stroke	
☐	Yes
☐	Yes, in 24hrs
☐	No
☐	After 2 weeks

Was decompressive craniectomy performed	
☐	Yes
☐	No

Atrial fibrillation/ flutter (AF)	
☐	Known AF
☐	Detected during hospitalization
☐	No AF detected
☐	Not screened
☐	Unknown

Stroke etiology (select all that apply)

☐	Large Artery Atherosclerosis (e.g., Carotid, or basilar stenosis)
☐	Cardioembolism (e.g., AF/flutter/ prosthetic heart valve)
☐	Stroke of other determined etiology (dissection, vasculopathy or hematologic disorder)
☐	Cryptogenic Stroke (Stroke of undetermined etiology including ESUS)
☐	Small Vessel Disease /Lacuna

Was CT/MR performed after IVT/MT?

☐ Yes, CT ☐ Yes, MR ☐ No

(Yes, CT / Yes, MR → Findings on CT/MRI after IVT/MT)

Findings on CT/MRI after IVT/MT (select all that apply)

☐	Brain infarct
☐	No bleeding
☐	Remote bleeding in the brain
☐	Bleeding at the site of infarction haemorrhagic infarction H I type 1
☐	Bleeding at the site of infarction haemorrhagic infarction H I type 2
☐	Bleeding at the site of infarction parenchymal haemorrhage PH type 1
☐	Bleeding at the site of infarction parenchymal haemorrhage PH type 2

POST ACUTE CARE (For patients in the hospital)

Temp checks (No. of times)	0	1	2	3	4+
Day 1	☐	☐	☐	☐	☐
Day 2	☐	☐	☐	☐	☐
Day 3	☐	☐	☐	☐	☐

In the first 72 hours of admission did patient develop fever of ≥ 37.5° C?

☐ Yes ☐ Yes ☐ Unknown

Was paracetamol or (other Antipyretic) administered for the first elevated temperature?

- ☐ Yes, within 1 hour of first elevated temperature
- ☐ Yes, after 1 hour of first elevated temperature
- ☐ No
- ☐ Contraindicated

Blood glucose level checks (no. of times)	0	1	2	3	4+
Day 1	☐	☐	☐	☐	☐
Day 2	☐	☐	☐	☐	☐
Day 3	☐	☐	☐	☐	☐

In the first 48 hours following admission did the patient develop a glucose level of greater or equal 10 mmols/L? {Y (mmol/l) = Xmg/ml/ 0.18}

☐ Yes ☐ Yes ☐ Unknown

Was insulin administered for the first elevated glucose (>=10 mmol/L)?

- ☐ Yes, within 1 hour of the first elevated glucose level
- ☐ Yes, after 1 hour of the first elevated glucose level
- ☐ No

Swallow screening performed

- ☐ Yes, within 4 hrs of admission
- ☐ Yes, within 24 hrs of admission
- ☐ Yes, after 24 hrs of admission
- ☐ Not done
- ☐ Not applicable (Patient intubated, NGS, etc.)

Which swallowing screening test performed

- ☐ Guss test
- ☐ Assist test
- ☐ Eat 10
- ☐ SVT
- ☐ SST
- ☐ Drinking water test
- ☐ Other (gag reflex not to be considered)

Who performed swallowing screening?

- ☐ Nurse
- ☐ Physician
- ☐ Other

Did patient successfully pass the swallow screening test?

- ☐ Yes
- ☐ No

Patient received physiotherapy?

- ☐ Yes
- ☐ No
- ☐ Not required

Patient received ergotherapy/ occupational therapy?

- ☐ Yes
- ☐ No
- ☐ Not required

Patient received by speech therapy

- ☐ Yes
- ☐ No
- ☐ Not required

Post stroke complications (select all that apply)

- ☐ Pneumonia
- ☐ Deep vein thrombosis (DVT)
- ☐ Pulmonary embolism
- ☐ Urinary tract infection
- ☐ Pressure sores
- ☐ Drip site sepsis
- ☐ Recurrence/extension of stroke
- ☐ Other
- ☐ None

DISCHARGE INFORMATION & TREATMENT (For patients hospitalized for > 48 hrs & patient transferred)

Discharge date	DD-MM-YYYY				
Modified Ranking Scale (MRS) score on discharge	1	2	3	4	1
	☐ Unknown				
NIHSS Score on discharge	Score				

Discharge destination	☐ Home
	☐ Transferred within the same centre
	☐ Transferred to another centre
	☐ Social care facility
	☐ Patient died

Treatment prescribed on discharge (select all that apply)	☐ Anti-diabetics	☐ Low molecular weight Heparin/Heparin
	☐ Anti-hypertensives	☐ Dabigatran
	☐ Aspirin (ASA)	☐ Rivoroxaban
	☐ Cilostazol	☐ Apixaban
	☐ Clopidogrel	☐ Edoxaban
	☐ Ticagrelol	☐ Anticoagulant not prescribed but recommended
	☐ Ticlopidine	☐ Statin
	☐ Prasugrel	☐ None
	☐ Dipyridamol, slow release	☐ Other
	☐ Warfarin	

Follow up appointment scheduled in your hospital for stroke management	☐ Yes
	☐ No, but recommended to schedule

If the patient was a smoker was he/she recommended a smoking cessation program	☐ Yes
	☐ No
	☐ Not a smoker

FOLLOW UP AFTER 3 MONTHS
(Only for patients getting discharged from hospital and not transferred patients)

Contact date	DD-MM-YYYY

Mode of Contact	☐ Telephonic/video (Patient or caregiver)
	☐ Visiting the outpatient clinic
	☐ Mobile application
	☐ Web application
	☐ Patient/care giver didn’t respond
	☐ Not contacted

3 Months Modified Ranking Scale (mRS) score	1	2	3	4	5
	6	Unknown			
